# Development of a conceptual model of early systemic sclerosis (scleroderma)

**DOI:** 10.1186/s13023-025-03917-8

**Published:** 2025-08-19

**Authors:** William R. Lenderking, Mary Kathleen Ladd, Nicola Barnes, Julia Braverman, Maria Gasior, Jutta Hofmann, John-Phillip Lawo, Dinesh Khanna

**Affiliations:** 1 PPD Evidera Patient-Centered Research, Thermo Fisher Scientific, Waltham, MA USA; 2 PPD Evidera Patient-Centered Research, Thermo Fisher Scientific, London, England; 3 CSL Behring, King of Prussia, PA USA; 4 CSL Innovation GmbH, Marburg, Germany; 5 University of Michigan, Ann Arbor, MI USA; 6 William Lenderking, PhD, PLLC, Harvard, MA, USA

**Keywords:** Conceptual disease model, Physical symptoms, Cutaneous systemic sclerosis, Scleroderma, Qualitative research, Concept elicitation interviews

## Abstract

**Background:**

Systemic sclerosis (SSc) is a rare connective tissue disorder with heterogeneous manifestations. Two predominant subtypes, limited cutaneous SSc (lcSSc) and diffuse cutaneous SSc (dcSSc), are distinguished based on skin involvement distribution. A comprehensive conceptual SSc model is needed to support measurement strategies for outcome studies. This qualitative study aimed to explore key SSc disease concepts and develop a conceptual disease model capturing the heterogeneous lived experiences of patients with SSc.

**Methods:**

Patient- and clinician-reported concepts specific to dcSSc (more severe and faster-progressing than lcSSc) were identified via a targeted literature review and used to develop a preliminary dcSSc symptom model and a semi-structured qualitative interview guide. The guide was used in concept elicitation interviews with adults with lcSSc and dcSSc. A final conceptual SSc symptoms model was refined based on interview results.

**Results:**

Disease concepts were retrieved from 35 peer-reviewed articles and 17 clinical trials focusing on patients with dcSSc. The preliminary dcSSc symptom model included skin, hand, gastrointestinal, pain, joint, muscle, mouth, sexual, lung, cardiovascular, cognitive, ocular, and other symptoms. During concept elicitation interviews, participants (n = 44) reported 112 unique symptoms (within 13 domains). Twenty-six symptoms had not previously been identified in pertinent literature. Hand and skin symptoms were reported by all participants. Over 95% of participants reported at least one gastrointestinal and pain symptom, around 80% reported joint and mouth symptoms, 70% reported muscle symptoms, and over 50% reported ocular symptoms. Cognitive, lung, sexual, and cardiac symptoms were reported by fewer than half of participants. Participants with dcSSc reported a broader variety of symptoms than those with lcSSc. However, concepts relevant to patients with dcSSc and lcSSc strongly overlapped, suggesting that a single conceptual model is appropriate to map symptoms for both subtypes. The overlap was further reflected in the most bothersome symptoms, which included skin fibrosis and hand symptoms for both populations.

**Conclusions:**

The final conceptual model captures the heterogeneous symptoms of SSc and reflects the lived experience of patients with SSc. It covers both clinical SSc subtypes and can support the choice and/or development of instruments to measure patient experiences in clinical trials.

**Supplementary Information:**

The online version contains supplementary material available at 10.1186/s13023-025-03917-8.

## Background

Systemic sclerosis (scleroderma, SSc) is a rare autoimmune connective tissue disorder affecting an estimated 7–44 per 100,000 individuals in Europe and North America [1]. SSc affects multiple organ systems and, due in part to a historical lack of disease-modifying therapies, is associated with high mortality rates [2, 3].

SSc pathogenesis is characterized by three pathological hallmarks: small vessel vasculopathy, autoantibody production, and widespread fibrosis, which all contribute to skin and internal organ damage [2, 4]. SSc phenotypic disease manifestations, progression rates, and outcomes are highly heterogeneous [2, 4–6]. The American College of Rheumatology/European League Against Rheumatism (ACR/EULAR) SSc classification criteria (for inclusion of SSc patients in clinical studies) include skin thickening of the fingers of both hands extending proximal to the metacarpophalangeal joints (this alone is sufficient for the patient to be classified as having SSc), skin thickening of other areas of the fingers, puffy fingers, fingertip lesions, telangiectasia, abnormal nailfold capillaries, interstitial lung disease and/or pulmonary arterial hypertension, Raynaud’s phenomenon, and SSc-related autoantibodies [7].

While SSc diagnosis should go beyond these classification criteria [8], it can be confounded by the sheer heterogeneity of SSc symptoms. Varying degrees of skin, lung, renal, cardiac, pulmonary, gastrointestinal, and musculoskeletal damage lead to unique clinical presentations (and correspondingly unique patient needs) [2].

Two main SSc subtypes are distinguished clinically based on the distribution of skin involvement [5, 9]. Patients with limited cutaneous SSc (lcSSc) experience localized skin thickening distal to the elbows and knees with or without face and neck involvement. Patients with diffuse cutaneous SSc (dcSSc) have both distal and proximal involvement of skin, including chest and abdomen, and experience rapid and widespread skin thickening in early disease stages and are at high risk of subsequent internal organ involvement, e.g., development of interstitial lung disease [6, 10].

SSc can result in disability and is associated with reduced health-related quality of life (HRQoL), impacting various physical, psychological, and social domains [11, 12]. However, owing to its rarity and heterogeneity, not only the classification and diagnosis but also the treatment of SSc can be challenging [4].

Difficulties in treating SSc and in assessing treatment efficacy are likely compounded by a lack of tools that comprehensively measure the heterogeneous symptoms of SSc. Indeed, a lack of valid trial endpoints—which are meaningful to both patients and healthcare professionals and capture the myriad clinical dimensions of SSc—may have biased recent clinical trials that failed to detect significant differences in specific primary endpoints [2, 13–15].

Conceptual disease models can provide a framework from which relevant aspects of a disease can be easily conveyed and understood. They can help identify relevant clinical endpoints and—by incorporating patient-reported concepts—ensure that symptoms or impacts considered important to the patient are assessed in clinical research trials and clinical practice. Recent Food and Drug Administration (FDA) guidance recommends a conceptual model as a necessary first step for justifying the clinical outcome assessment (COA) strategy in clinical trials [16].

A comprehensive SSc symptom model is needed to support comprehensive, minimally burdensome, and sensitive measurement strategies for evaluations in clinical trials, and for the development of future COAs. No such model currently exists in pertinent peer-reviewed literature.

This article outlines the development of a conceptual SSc symptom model based on a targeted literature review and concept elicitation interviews with patients. The primary objective was to summarize key SSc disease concepts and capture the myriad patient experiences of SSc in a conceptual SSc symptom model. Additionally, this study aimed to assess whether the two subtypes, dcSSc and lcSSc, exhibit sufficient differences from the patient perspective to necessitate distinct subtype-specific symptom models, or if a single unified model can adequately represent both subtypes (secondary objective).

## Methods

The study was performed in two steps. In step 1, a targeted literature review was conducted to identify and synthesize key patient- and clinician-reported dcSSc concepts. It served to develop a preliminary symptom dcSSc model and a semi-structured qualitative interview guide. In step 2, the semi-structured interview guide was used in concept elicitation interviews with adults with SSc. The final symptom model was refined based on patient interview results to generate an in-depth understanding of the SSc disease experience.

## Step 1: Targeted literature review and preliminary conceptual model development

### Targeted literature review

A targeted literature review served to identify and synthesize key patient- and clinician-reported dcSSc concepts (symptoms and impacts). The authors initially focused on dcSSc as it is generally more severe and progresses more quickly than lcSSc. The literature reviewed covered FDA and European Medicines Agency (EMA) label claims for patient-reported outcomes (PROs) for use in SSc patients, PROs used as endpoints in SSc clinical trials, and peer-reviewed publications.

### FDA and EMA label claims

FDA and EMA databases (https://www.accessdata.fda.gov/scripts/cder/daf/index.cfm; https://www.ema.europa.eu/en/medicines) were searched in January 2020 for product labels citing COAs of drugs approved for SSc using the search terms “systemic sclerosis” or “scleroderma”.

### Outcome assessments used as *endpoints* in SSc clinical trials

US and European clinical trials databases (clinicaltrials.gov and clinicaltrialsregister.eu) were searched in January 2020 to identify trials in dcSSc and SSc that included COAs as primary, secondary, or exploratory endpoints. The search strategy is detailed in Supplementary Table S1. Data on study title, phase, objective, population, COA utilized, design, primary and secondary outcomes, identifier, and sponsor were extracted into a Microsoft Excel® template.

### Peer-reviewed publications

Ovid MEDLINE®, PsycINFO, and Cochrane database searches were performed on 09 March 2020 using a combination of the following search terms: *Population (keywords related to SSc and dcSSc) AND Methods (keywords related to qualitative research OR to clinical outcomes assessment instruments OR to impact, burden, or HRQoL).* The search was limited to peer-reviewed articles focused on a population with a diagnosis of dcSSc or SSc, published in English between 2012 and 2020 and excluded articles focusing primarily on nonhuman studies, editorials, conference abstracts, white papers, case studies, and letters to the editor. For inclusion, articles had to include at least one COA instrument not identified in the labels search or the clinical trials search OR use qualitative research methods to obtain patient experience. Articles were excluded if no abstract or full text was available. A full list of search criteria is provided in Supplementary Tables S2 and S3.

Search results were screened in a two-level process (first level: titles and abstracts screened; second level: full-text articles screened), and articles were excluded if they failed to meet the criteria detailed above. Two Evidera researchers reviewed the titles and abstracts, and a third confirmed the selected abstracts and reviewed any initially rejected. Relevant conceptual information was extracted from the full-text articles into a Microsoft Excel® template. Data from peer-reviewed publications were extracted on study design, objectives, sample size, eligibility criteria, concepts mentioned, concepts reported, COA instruments used, and relevant results.

### Preliminary conceptual model and interview guide development

Concepts identified via the targeted literature review (i.e., retrieved from label claims, COAs, and peer-reviewed literature) were categorized into dcSSc symptoms and impacts. Data pertaining to dcSSc symptoms were used to inform a preliminary dcSSc symptom model. In turn, this model was used to develop a semi-structured interview guide for qualitative patient interviews. Questions in the guide were designed to probe participants on symptoms included in the preliminary model.

## Step 2: Cross-sectional, qualitative research study and conceptual model refinement

### Study design

This was a cross-sectional, qualitative research study involving one-on-one, qualitative, concept elicitation interviews.

### Ethics

The study protocol, informed consent procedures, and participant materials were approved by the Advarra institutional review board (Columbia, Maryland, USA). Written informed participant consent was obtained prior to participation in the study.

### Study setting and participants

Participants were recruited from ten rheumatology practices in the US (Supplementary Table S4). The practices reached out to patients during clinic visits or called potentially eligible patients based on chart review.

Eligible participants were English-speaking adults (≥ 18 years old) with documented ACR/EULAR classification criteria for SSc [7] and a disease duration of ≤ 5 years (defined as the time from the first clinician-reported non-Raynaud’s phenomenon manifestation). Early SSc participants were recruited as ongoing current trials generally focus on early SSc (defined as first 5–7 years after onset of non-Raynaud’s phenomena). For participants with dcSSc, additional inclusion criteria were a modified Rodnan skin score ≥ 15 or skin thickening in some areas on the face fingers, hands, forearms, feet or legs and at least 2 areas involving upper arms, chest, abdomen, and thighs. Furthermore, participants with dcSSc whose disease duration was > 18 months to ≤ 5 years had to have experienced a tendon friction rub or at least one new area affected in the past 6 months. For participants with lcSSc, an additional inclusion criterion was skin involvement limited to fingers, hands, forearms, feet and legs with or without face. Arms, torso and thighs were not to be affected.

Participants were excluded from the study if they had a primary rheumatic autoimmune disease other than SSc or any other condition that could potentially compromise HRQoL and interfere with interpretation of elicited concepts.

### Interview procedures

Ninety-minute phone interviews encompassing questions on sclerosis-associated symptoms and basic demographic information were conducted by trained interviewers in English. Interviewers used the qualitative semi-structured guide developed in step 1 to ensure consistency among interviews. Interviewers posed open-ended questions to elicit participants’ insights into SSc symptoms. Participants were encouraged to elaborate upon their SSc symptoms and to list their three most bothersome symptoms.

At the end of each interview, participants completed the sociodemographic and clinical questionnaire they had been sent before the interview while remaining on the phone with the interviewer. All interviews were audio-recorded, transcribed, and deidentified via removal of all protected health and non-health-related personal information. Patients received an incentive in line with fair market value for their participation in the interview.

### Quantitative data analysis (sociodemographic and clinical questionnaire; PRO measures)

Quantitative data were curated using a direct fax-to-computer data management system (DFdiscover). Descriptive statistics were calculated. No form of artificial intelligence was used to code or qualitatively analyze the interview data.

### Qualitative data analysis

Data were coded and analyzed following a content analysis approach. A coding dictionary was derived from the interview guide and was modified and updated throughout the coding process. A preliminary coding framework was developed and used to code the first interview transcript by three coders; the coders then participated in a coding resolution meeting with the study team. During this meeting, the team reviewed the agreement across coders, and the coding framework was revised (via addition of codes and clarification of code comments). The team discussed the rationale for which codes were used, determined the appropriate codes to use where there were discrepancies, and updated the guidance/definitions for use of these codes to ensure clarity. The coding team was trained on the refined codebook and proper code application for the remaining transcripts. The coders then coded the remaining transcripts independently in a split-coding approach. As new codes were identified, they were iteratively added to the coding dictionary. One senior reviewer conducted a quality check of all coded data to ensure codes were applied accurately, consistently, and comprehensively.

Qualitative data (notes, transcripts, and audio recordings) were systematically analyzed using ATLAS.ti^©^. Concept saturation relating to SSc symptoms was assessed after the final interview. Interview transcripts were organized in order of completion. Once no new concepts appeared in the transcripts, concept saturation was considered achieved (12).

### Conceptual model refinement

The preliminary conceptual model of physical SSc symptoms was revised to account for symptoms newly identified during the interviews, symptoms not endorsed during the interviews, and the inclusion of patient-friendly language. The model was updated to indicate which symptoms were reported only by one SSc subtype (dcSSc or lcSSc).

## Results

### Step 1: Targeted literature review and preliminary conceptual model development

#### Targeted literature review

##### FDA and EMA label claims

The label claims search identified three COA instruments in two FDA labels for products mentioning SSc. Both labels were for products in primary pulmonary hypertension and referenced SSc in the label description. The three COA instruments mentioned therein were not specific to SSc. The search did not identify any labels for SSc in the EMA database.

##### Outcome assessments used as endpoints in SSc clinical trials

The clinical trials search and screening yielded 17 studies focusing on patients with dcSSc. Nineteen COA instruments were identified as dcSSc and SSc endpoints in these clinical trials; seven were clinician-reported outcomes, and 12 were PROs (Supplementary Table S5).

##### Peer-reviewed publications

The database searches identified 426 results for review. After title and abstract screening, 34 references were included for full-text review. One additional article (not captured by the search due to its EMBASE indexing status) was suggested by the study sponsor CSL Behring, screened, and included in the full-text review. Thirty-five full-text articles were thus judged to be relevant and were included in the review. The full Preferred Reporting Items for Systematic Reviews and Meta-Analyses (PRISMA) diagram can be found in Supplementary Fig. S1.

The 35 full-text articles comprised 27 quantitative studies, four qualitative studies, two mixed methods studies, and two reviews. Thirty studies involved patients with SSc; 14 of these studies included patients with dcSSc, and 13 included patients with lcSSc. The studies involved patients recruited from over 60 countries. A further 38 COA instruments were identified (Supplementary Table S5).

### Preliminary symptom model development

A preliminary symptom model captured the heterogeneity of dcSSc symptoms (Supplementary Fig. S2). It included skin, hand, gastrointestinal, pain (including neuropathic pain), joint, muscle, mouth, sexual, lung, cardiovascular, cognitive, ocular, and other symptoms.

## Step 2: Cross-sectional, qualitative research study and conceptual model refinement

### Participant demographics

Of the 44 US-based participants participating in the semi-structured concept elicitation interviews, 32 were diagnosed with dcSSc, and 12 were diagnosed with lcSSc (Table 1). The mean age of the total sample was 51.7 years, and age ranged from 18 to 75 years. Eleven participants were male (25%), and 33 were female (75%). Most participants were White (71.8%) and married or in a relationship (64.1%). 38.5% of participants were employed full- or part-time, 25.6% were retired, and 20.5% were disabled. Five participants did not return their sociodemographic forms. For these patients, race and ethnicity, marital status, employment, and education were kept as missing.

**Table 1:** Participant-reported sociodemographic characteristics

	Study Population (N = 44^1^)	DcSSc (N = 32)	LcSSc (N = 12)
*Age*			
Mean (SD)	51.7 (13.6)	49.1 (13.6)	58.8 (11.1)
Median [min, max]	54.0 [18.0–75.0]	48.5 [18.0–69.0]	59.5 [38.0–75.0]
*Biological Sex, n* (%)			
Male	11 (25.0)	10 (31.3)	1 (8.3)
Female	33 (75.0)	22 (68.8)	11 (91.7)
Ethnicity, n (%)			
Hispanic or Latino	8 (20.5)	5 (17.9)	3 (27.3)
Not Hispanic or Latino	18 (46.2)	13 (46.4)	5 (45.5)
Other^2^	13 (33.3)	10 (35.7)	3 (27.3)
*Race, n* (%)			
White	28 (71.8)	18 (64.3)	10 (90.9)
Black or African American	2 (5.1)	2 (7.1)	0
Asian	3 (7.7)	3 (10.7)	0
Other^3^	6 (15.4)	5 (17.9)	1 (9.1)
*Marital Status, n* (%)			
Married	19 (48.7)	13 (46.4)	6 (54.5)
Single	10 (25.6)	10 (35.7)	0
Divorced/separated	2 (5.1)	1 (3.6)	1 (9.1)
Widowed	2 (5.1)	0	2 (18.2)
Single in a relationship	6 (15.4)	4 (14.3)	2 (18.2)
*Employment Status, n* (%)			
Employed, full-time	11 (28.2)	10 (35.7)	1 (9.1)
Employed, part-time	4 (10.3)	4 (14.3)	0
Homemaker	2 (5.1)	0	2 (18.2)
Unemployed	3 (7.7)	3 (10.7)	0
Retired	10 (25.6)	5 (17.9)	5 (45.5)
Disabled	8 (20.5)	5 (17.9)	3 (27.3)
Other^4^	1 (2.6)	1 (3.6)	0
*Education, n* (%)			
Secondary/high school	8 (20.5)	5 (17.9)	3 (27.3)
Some college	9 (23.1)	7 (25.0)	2 (18.2)
College degree	10 (25.6)	7 (25.0)	3 (27.3)
Postgraduate degree	9 (23.1)	7 (25.0)	2 (18.2)
Technical or vocational degree	3 (7.7)	2 (7.1)	1 (9.1)

^1^Five participants did not return their sociodemographic forms. For age and biological sex, the denominators are the full cohort (as specified in the column header). For all other items, the n’s are study population = 39, dcSSc = 28, and lcSSc = 11

^2^Other ethnicity responses included Asian (n = 1), Asian/Korean (n = 1), Black American (n = 1), Greek (n = 1), Italian (n = 1), Lebanese (n = 1), South East Asian (n = 1), White (n = 3), and did not specify (n = 3)

^3^Other race responses included Lebanese (n = 1); Mexican (n = 1); White, Black, and Asian (n = 1); and did not specify (n = 3)

^4^The other employment response was on leave from full-time employment due to scleroderma complications (n = 1)

Abbreviations: dcSSc, diffuse cutaneous systemic sclerosis; lcSSc, limited cutaneous systemic sclerosis; SD, standard deviation

### Participant interviews—saturation

Overall, concept saturation was reached after 34 interviews. For dcSSc, saturation was reached after the 22nd interview. For lcSSc, saturation was not reached, as three new sexual symptoms (dyspareunia or painful intercourse, impaired arousal, and impaired orgasm) and two new cognitive symptoms (language and brain fog) were endorsed or spontaneously reported during the 11th interview with a participant with lcSSc.

### Participant interviews—symptoms

In total, 112 unique symptoms (within 13 symptom domains) were reported by participants during the concept elicitation interviews, 26 of which were not previously identified during the targeted literature review (these are designated as “newly reported” throughout the text). Supplementary Table S6 lists all patient-reported symptoms. Supplementary Table S7 provides patient quotes illustrating the most common symptoms across subtypes. Patients were asked about their symptoms; however, a number of patients also reported signs and/or diagnoses, referring to these as symptoms. In this section, we maintain the symptom nomenclature, but distinguish between signs, symptoms and diagnoses in the symptom model.

At least one hand and skin symptom each was reported by all study participants. The most frequently reported hand symptom was Raynaud’s phenomenon/cold intolerance (95.5% overall; 93.8% dcSSc; 100% lcSSc), followed by loss of grip strength (70.5%; 78.1% dcSSc; 50% lcSSc), loss of hand function (61.4% overall; 78.1% dcSSc; 33.3% lcSSc), and puffy fingers (50% overall; 46.9% dcSSc; 58.3% lcSSc). Amputation was spontaneously reported by one participant with dcSSc (3.1%) and one with lcSSc (8.3%).

The following skin symptoms were most frequently reported and were more often reported by participants with dcSSc than by those with lcSSc: skin fibrosis (81.8% overall; 93.8% dcSSc; 50.0% lcSSc), skin color change (72.7% overall; 75.0% dcSSc; 66.7% lcSSc), skin itchiness (70.5% overall; 78.1% dcSSc; 50.0% lcSSc), and skin swelling (54.5% overall; 59.4% dcSSc; 41.7% lcSSc). These symptoms were more often reported by participants with dcSSc than by those with lcSSc. Dry skin (n = 10 (22.7%) overall; n = 4 (12.5%) dcSSc; n = 6 (50%) lcSSc) and red skin (n = 9 (20.5%) overall; n = 5 (15.6%) dcSSc; n = 4 (33.3%) lcSSc) were two skin symptoms not previously identified in the literature (and consequently not included in the initial conceptual model) and were more frequently reported by participants with lcSSc than by those with dcSSc. Easy bruising was newly reported by one participant with dcSSc (3.1%) but not by any participant with lcSSc.

Gastrointestinal symptoms were reported by most participants (95.5% overall), with 30 participants with dcSSc (93.8%) and 12 participants with lcSSc (100.0%) reporting at least one such symptom. Participants in both groups were affected by gastrointestinal symptoms related to esophageal involvement (heartburn/reflux, difficulty swallowing, nausea) and intestinal involvement (diarrhea, constipation, distention/bloating). Across subtypes, the most frequently reported gastrointestinal symptoms were acid heartburn/reflux (84.1% overall; 84.4% dcSSc; 83.3% lcSSc), and difficulty swallowing (50% overall; 43.8% dcSSc; 66.7% lcSSc). Gas was not previously identified in the literature, but was reported by three participants (6.25% dcSSc; 8.3% lcSSc). Early satiety and gastrointestinal bleeding, two gastrointestinal symptoms not previously identified in the literature (and consequently not included in the initial conceptual model), were reported by participants with dcSSc (n = 2 (6.3%) each).

Pain symptoms were also reported by 95.5% of participants (96.9% dcSSc; 91.7% lcSSc). The most frequently reported pain symptoms were joint pain (72.7% overall; 78.1% dcSSc; 58.3% lcSSc) and hand pain (56.8% overall; 56.3% dcSSc; 58.3% lcSSc). Two previously unidentified pain symptoms, foot pain alternately attributed to Raynaud’s phenomenon or exertion (n = 6 (13.6%) overall; n = 4 (12.5%) dcSSc; n = 2 (16.7%) lcSSc) and headaches (n = 2 (6.3%) dcSSc), were spontaneously reported.

Mouth symptoms, reported by 81.8% of participants, were more often reported by participants with dcSSc (93.8%) than by those with lcSSc (50.0%). Dry mouth was the most commonly reported mouth symptom (52.3% overall; 53.1% dcSSc; 50.0% lcSSc), with many participants experiencing dry mouth upon awakening during the night. Three mouth symptoms, numbness of the mouth, tightness of the tongue, and mouth sores, not previously reported in pertinent literature were each reported by two participants (overall: n = 2 (4.5%)).

Joint symptoms (79.5% overall) were reported by most participants with dcSSc (90.6%) and over half of participants with lcSSc (58.3%). The most frequently reported joint symptom was stiffness (45.5% overall; 50% dcSSc; 33.3% lcSSc) in fingers, wrists, elbows, shoulders, knees, hips, and ankles.

Muscle symptoms (70.5%) were more commonly reported by participants with dcSSc (75.0%) than by those with lcSSc (58.3%). The most commonly reported muscle symptom was muscle weakness (56.8% overall; 62.5% dcSSc; 41.7% lcSSc). Five previously unidentified muscle symptoms were reported. Three of these, tendon tightness (n = 2 (6.3%)), muscle tightness (n = 3 (9.4%)), and muscle atrophy (n = 1 (3.1%)), were reported by participants with dcSSc; two, tremors and muscle cramps, were reported by patients with lcSSc (n = 1 (8.3%) each).

Ocular symptoms were reported by half of the participants (50.0% overall; 53.1% dcSSc; 41.7% lcSSc). Several eye conditions included in the initial conceptual model were not endorsed by study participants (e.g., eye web growth, growth on conjunctiva). Dry eye, the most frequently reported ocular symptom overall, was newly reported by study participants. It was only reported by participants with dcSSc (19.2% overall; 25.0% dcSSc). Sensitivity to light and vision impairment were each newly reported by six participants overall (13.6%), the majority of whom were participants with dcSSc (only one participant with lcSSc reported vision impairment).

Cognitive symptoms were reported by nearly half of the participants (47.7% overall; 53.1% dcSSc; 33.3% lcSSc). The most frequently reported cognitive symptom was difficulty remembering (29.5% overall; 31.3% dcSSc; 25.0% lcSSc). Brain fog was spontaneously reported by five participants (11.4% overall; 12.5% dcSSc; 8.3% lcSSc). This symptom was not previously included in pertinent literature. Participants with dcSSc reported more cognitive symptoms than those with lcSSc.

Lung and sexual symptoms were each reported by 45% of participants. The occurrence of lung symptoms was similar between subtypes (46.9% dcSSc; 41.7% lcSSc). The most frequently reported lung symptom was dyspnea (36.4% overall; 40.6% dcSSc; 25.0% lcSSc). More participants with dcSSc (59.4%) endorsed sexual symptoms than those with lcSSc (16.7%). The most frequently reported sexual symptom was lack of desire (25.0% overall; 28.1% dcSSc; 16.7% lcSSc).

Cardiac symptoms were not often reported (29.5% overall) but were endorsed more often by participants with dcSSc (34.4%) than those with lcSSc (16.7%). The most common cardiac symptom, edema (20.4% overall; 25% dcSSc; 8.3% lcSSc), was endorsed by an lcSSc patient (n = 1) and irregular heartbeat was endorsed by one lcSSc patient.

The most common miscellaneous symptoms (i.e., those that do not fit the previous categories) were fatigue (79.5% overall; 87.5% dcSSc; 58.3% lcSSc), weight loss (50% overall; 62.5% dcSSc; 16.7% lcSSc), and changes in appearance (45.5% overall; 56.3% dcSSc; 16.7% lcSSc). Two previously unidentified miscellaneous symptoms, poor circulation (n = 2 (4.5%) overall; n = 1 (3.1%) dcSSc; n = 1 (8.3%) lcSSc) and numbness (n = 1 (8.3%) lcSSc), were reported.

Table 2 summarizes the most frequently reported symptoms (reported by ≥ 50% of patients) by SSc subtype; 10 of the most frequently reported symptoms were the same across subtypes.

**Table 2:** Most frequently reported symptoms by SSc subtype

dcSSc (n = 32)		lcSSc (n = 12)	
Symptom	Count, n (%)	Symptom	Count, n (%)
*Skin fibrosis*	30 (93.8)	*Raynaud’s phenomenon/cold intolerance*	12 (100.0)
*Raynaud’s phenomenon/cold intolerance*	30 (93.8)	*Heartburn/Reflux*	10 (83.3)
*Fatigue*	28 (87.5)	*Skin color change*	8 (66.7)
*Heartburn/Reflux*	27 (84.4)	Difficulty swallowing	8 (66.7)
*Loss of grip strength*	25 (78.1)	*Hand pain*	7 (58.3)
*Joint pain*	25 (78.1)	*Joint pain*	7 (58.3)
*Skin itchiness*	25 (78.1)	Puffy fingers	7 (58.3)
*Skin color change*	24 (75.0)	*Fatigue*	7 (58.3)
Loss of hand function	23 (71.9)	*Loss of grip strength*	6 (50.0)
Weight loss	20 (62.5)	*Skin itchiness*	6 (50.0)
*Muscle weakness*	20 (62.5)	*Muscle weakness*	6 (50.0)
Skin swelling	19 (59.4)	*Skin fibrosis*	6 (50.0)
Difficulty opening mouth	18 (56.3)	Dry skin	6 (50.0)
Changes in appearance	18 (56.3)	Dry mouth	6 (50.0)
*Hand pain*	18 (56.3)		

dcSSc, diffuse cutaneous systemic sclerosis; lcSSc, limited cutaneous systemic sclerosis

Italics indicate the most frequently reported symptoms that were the same across subtypes

Thirty-four symptoms were listed as most bothersome symptoms by participants (note: some participants reported only one or two most bothersome symptoms rather than the requested three). Across SSc subtypes, the most bothersome symptom was skin fibrosis (Table 3). Several aspects of skin fibrosis, including its severity, were described as particularly bothersome:

*“The tightening of the skin, the thickening of the skin, the range of motion that I’ve lost in my hands and fingers. That’s absolutely the most bothersome stuff”. 007-003 (dcSSc)*

*“When I wasn’t on meds, my skin was so tight that my skin on my fingers…it was like the best way I could describe it is if you squash a grape, like how it bursts? That’s how my skin would pop from how bad the tightness was”. 014-003 (lcSSc)*

**Table 3:** Most bothersome participant-reported SSc symptoms

Symptom	Overall count	dcSSc, n (%)	lcSSc, n (%)
Skin fibrosis	20	16 (80.0)	4 (20.0)
Loss of hand function	12	11 (91.7)	1 (8.3)
Raynaud’s phenomenon/cold intolerance	9	3 (33.3)	6 (66.7)
Fatigue	7	7 (100.0)	0
Dyspnea	6	5 (83.3)	1 (16.7)
Heartburn/Reflux	5	4 (80.0)	1 (20.0)
Joint contracture	5	4 (80.0)	1 (20.0)
Joint pain	5	3 (60.0)	2 (40.0)
Muscle weakness	4	3 (75.0)	1 (25.0)
Skin itch	4	3 (75.0)	1 (25.0)
Hand pain	4	3 (75.0)	1 (25.0)
GI symptoms	4	3 (75.0)	1 (25.0)
Change in appearance	3	2 (66.7)	1 (33.3)
Hand swelling	3	2 (66.7)	1 (33.3)
Skin ulcers	3	2 (66.7)	1 (33.3)
Difficulty opening mouth	3	3 (100.0)	0
Difficulty swallowing	3	1 (33.3)	2 (66.7)
Loss of range of motion	3	3 (100.0)	0
Muscle pain	2	2 (100.0)	0
Skin sensitivity	2	0	2 (100.0)
Joint stiffness	2	2 (100.0)	0
Brain fog	1	1 (100.0)	0
Joint inflammation	1	1 (100.0)	0
Chronic pain	1	1 (100.0)	0
Pulmonary hypertension	1	0	1 (100.0)
Weight loss	1	1 (100.0)	0
Erectile dysfunction	1	1 (100.0)	0
Skin itching	1	1 (100.0)	0
Attention	1	1 (100.0)	0
Skin swelling	1	1 (100.0)	0
Losing vision	1	0	1 (100.0)
Tremors	1	0	1 (100.0)
Grip strength	1	1 (100.0)	0
Skin infections	1	0	1 (100.0)

Participants were asked to report their three most bothersome symptoms; some participants reported only one or two most bothersome symptoms

Abbreviations: dcSSc, diffuse cutaneous systemic sclerosis; GI, gastrointestinal; lcSSc, limited cutaneous systemic sclerosis; SSc, systemic sclerosis

The most bothersome symptoms for dcSSc participants included skin fibrosis, loss of hand function, and fatigue. One participant who described fatigue as their most bothersome symptom stated the following:

*“I never used to get tired, I mean, I was in the service and my daily runs were 12-mile runs every morning. I was young then, okay, but still I could run 12 miles and I’d never get tired. So that’s part of it, the fatigue, the weakness”. 004-004 (dcSSc)*

The most bothersome symptoms for lcSSc participants included Raynaud’s phenomenon/cold intolerance and skin fibrosis. One participant described their Raynaud’s phenomenon/cold intolerance as follows:

*“My hands (…) get so cold and they hurt, and whenever they’re that way, I can’t use them. I can’t even get my wallet out of my back pocket whenever my hands are that way”. 012-001 (lcSSc)*

### Model revision and final model

**Fig. 1:**
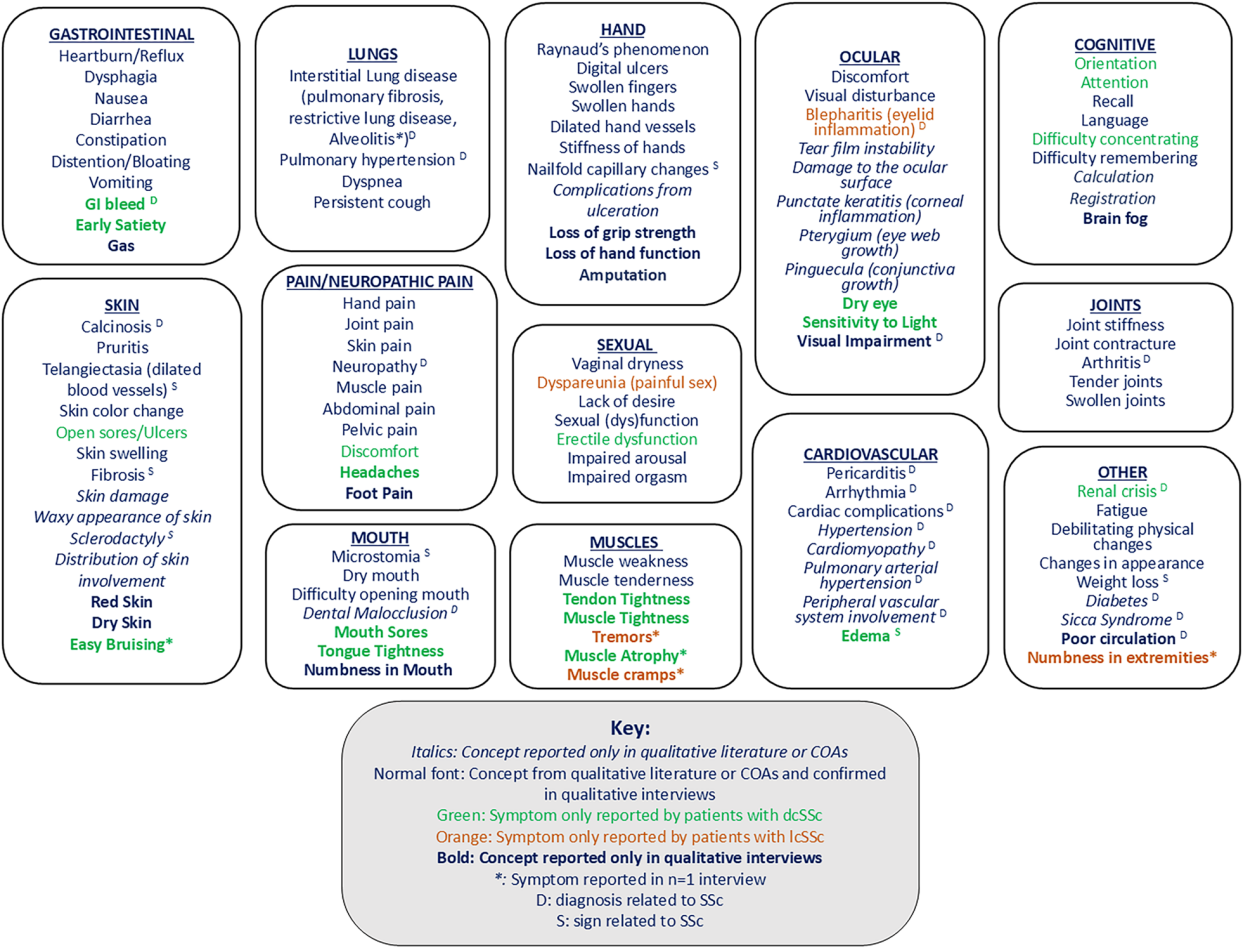
Conceptual model of the physical symptoms of systemic sclerosis. Abbreviations: COA, clinical outcome assessment; GI, gastrointestinal

The preliminary model of SSc symptoms was revised to account for the 26 symptoms newly identified during the interviews, for symptoms not endorsed during the interviews, for the inclusion of patient-friendly or lay language, and for the symptoms reported only by patients with one subtype (dcSSc, 19 symptoms; lcSSc, 5 symptoms). In the model, wording from concepts identified in the literature review has been retained, and patient wording used for concepts reported only in the interviews. The final model, providing a comprehensive representation of symptoms experienced by people living with SSc, is presented in Fig. 1. Signs and diagnoses, reported by some patients as symptoms (e.g., pulmonary hypertension), are identified in the model. Some concepts were merged, when patients used different terms to identify the same concept: reflux and heartburn were merged; a number of lung diagnoses (pulmonary fibrosis, restrictive lung disease and alveolitis) were merged under interstitial lung disease; and dilated blood vessels were merged with telangiectasia.

## Discussion

A conceptual SSc symptom model was developed based on insights from a targeted literature review (step 1) and qualitative concept elicitation interviews with patients (step 2). We identified 112 unique SSc symptoms within 13 symptom domains. Across SSc subtypes, both skin and hand symptoms were reported by all participants; over 90% of all participants reported at least one gastrointestinal and pain symptom, over 70% reported joint symptoms, and over 50% reported muscle, mouth, and sexual symptoms.

While overall symptom frequencies are consistent with past findings—e.g., skin and hand symptoms are so common as to feature as ACR/EULAR SSc classification criteria (7), and gastrointestinal tract involvement is known to occur in nearly all patients with SSc [17]—ten symptom domains within the conceptual model included 26 unique symptoms not identified in our targeted literature review. These 26 symptoms were newly reported by study participants.

Certain newly reported symptoms likely reflect subtle variations of perception or phrasing of previously known symptoms. Thus, e.g., within the skin symptom domain, dry and red skin may represent manifestations of skin fibrosis. Similarly, within the gastrointestinal domain, bleeding may be linked to known symptoms such as diarrhea [17]; within the muscle domain, muscle tremors may derive from muscle weakness [18]; and within the hand domain, amputation may be a consequence of Raynaud’s phenomenon [2]. Including these distinctions might nevertheless be useful to patients and clinicians alike and provides a more nuanced understanding of SSc disease concepts. Other newly reported symptoms, such as mouth sores or numbness, represent distinct novel SSc symptoms and are important additions to this conceptual model. When relevant, symptoms that patients reported with different terms, but which are conceptually or clinically the same, have been merged. This ensures that the concepts important to patients are retained, and that the model remains clinically relevant and clear.

Within the ocular domain, several symptoms were not endorsed by study participants. There are limited proven associations between SSc and ocular abnormalities [19]. However, dry eye symptoms have previously been associated with SSc.

Symptoms were generally more frequently reported by dcSSc than lcSSc patients (with the notable exception of Raynaud’s phenomenon/cold intolerance: 93.8% dcSSc versus 100.0% lcSSc). However, the concepts relevant to patients with dcSSc and lcSSc almost completely overlapped, suggesting that a single conceptual model is appropriate to map symptoms in patients with either condition: ten of the most frequently reported symptoms were the same across subtypes.

The overlap between SSc subtypes was further reflected in the most bothersome symptoms, which included skin fibrosis and hand symptoms (dcSSc: loss of hand function; lcSSc: Raynaud’s phenomenon/cold intolerance) for both participant populations.

The conceptual model includes mapping against existing COAs and identifies gaps in every one of the 13 symptom domains, showing that a single COA is currently inadequate to accurately cover concepts of importance to patients with SSc.

Concept elicitation methods allow concepts to emerge spontaneously by asking open-ended questions in a non-leading manner. Understanding SSc symptoms from the patient’s perspective is crucial to ensuring that conceptual disease models—and PROs based on them—have meaning and relevance to the population of interest [20]. This aligns with the FDA’s guidance on patient-focused drug development, which highlights the importance of capturing the patient voice within clinical research [16]. Qualitative methods provide a nuanced understanding of disease concepts and are increasingly recognized as adding value to (quantitative) SSc research [21]. The SSc symptom model developed based on the literature and informed by the qualitative interviews, will be critical to help researchers identify the most important symptoms to assess in clinical trials, and to inform the selection, validation and development of COAs to measure those symptoms. The patients’ perception remains the key driver for content in this model; concepts that were reported as symptoms by patients, but that from a clinical perspective are considered signs or diagnoses have been identified as such.

The fact that overall concept saturation was not reached until the 34th interview reinforces the heterogeneity of SSc. While saturation was achieved (albeit late) for the full study sample, saturation was not achieved within both individual subtypes, presenting a limitation of this study. Saturation was not reached for the lcSSc population, indicating that the sample size may have been too small to capture low-prevalence disease concepts in this subgroup. Our preliminary symptom model and interview guide were developed based on a literature review predominantly targeting dcSSc concepts. This may have somewhat biased the elicitation of lcSSc concepts during the interviews. Given the clear overlap between subgroups, a comprehensive understanding of lcSSc concepts may nevertheless have been reached.

The interviews were conducted only with English-speaking participants based in the US; our findings may thus not necessarily represent the lived experience of SSc patients accustomed to other cultures and health systems, although the predominantly White study population reflected different diverse cultural subgroups. Future research into the international patient experience could explore linguistic and cross-cultural nuances of SSc.

SSc has been reported to predominantly affect women, with a reported female:male ratio of 3.8–11.5:1 in Europe and 4.6–15:1 in North America [1]. Diagnosis of SSc has been reported to occur at the age of 33.5–59.8 years in Europe and 46.1–49.1 years in North America) [1]. The predominantly female (75%) study population with a mean age of 51.7 years was therefore likely representative of the general patient population in terms of sex and age.

The overall study population included participants of multiple races but was weighted towards White participants (72%), limiting the generalizability of these findings. Given the fact that severe SSc disproportionately affects African American patient populations and varies in clinical presentation by race (African American patients are more likely to experience renal crises or progressive interstitial lung disease than White patients) [1, 2], further research should focus on elicitation of concepts in a more diverse population. We focus on early SSc as we wanted to develop a symptom model relevant to the subjects recruited in current clinical trials; the number of symptoms and bothersomeness of these symptoms may have differed in patients with late disease.

## Conclusions

Our conceptual model captures the highly heterogeneous physical symptoms of SSc. Since dcSSc and lcSSc were found to differ in degree rather than in kind, our model covers both clinical SSc subtypes. It reflects the lived experience of patients with SSc and may help researchers select and validate existing instruments or develop novel disease-specific PROs for use in future clinical SSc trials.

## Supplementary Information

Additional file 1.

## Data Availability

The datasets used and/or analyzed during the current study are available from the corresponding author on reasonable request.

## Abbreviations

ACR/EULAR: American College of Rheumatology/European League Against Rheumatism
COA: Clinical trials outcome assessment
dcSSc: Diffuse cutaneous systemic sclerosis
EMA: European Medicines Agency
FDA: Food and Drug Administration
HRQoL: Health-related quality of life
lcSSc: Limited systemic sclerosis
PRISMA: Preferred Reporting Items for Systematic Reviews and Meta-Analyses
PRO: Patient-reported outcome
SSc: Systemic sclerosis

## References

[1] Bergamasco A, et al. Epidemiology of systemic sclerosis and systemic sclerosis-associated interstitial lung disease. Clin Epidemiol. 2019;11:257–73. 31114386 10.2147/CLEP.S191418 PMC6497473

[2] Volkmann ER, Andreasson K, Smith V. Systemic sclerosis. Lancet. 2023;401(10373):304–18. 36442487 10.1016/S0140-6736(22)01692-0 PMC9892343

[3] Elhai M, et al. Mapping and predicting mortality from systemic sclerosis. Ann Rheum Dis. 2017;76(11):1897–905. 28835464 10.1136/annrheumdis-2017-211448

[4] Bukiri H, Volkmann ER. Current advances in the treatment of systemic sclerosis. Curr Opin Pharmacol. 2022;64: 102211. 35447517 10.1016/j.coph.2022.102211 PMC9466985

[5] Denton CP, Khanna D. Systemic sclerosis. Lancet. 2017;390(10103):1685–99. 28413064 10.1016/S0140-6736(17)30933-9

[6] Ferreli C, et al. Cutaneous manifestations of scleroderma and scleroderma-like disorders: a comprehensive review. Clin Rev Allergy Immunol. 2017;53(3):306–36. 28712039 10.1007/s12016-017-8625-4

[7] van den Hoogen F, et al. 2013 classification criteria for systemic sclerosis: an American College of Rheumatology/European League against Rheumatism collaborative initiative. Arthritis Rheum. 2013;65(11):2737–47. 24122180 10.1002/art.38098 PMC3930146

[8] Avouac J, et al. Preliminary criteria for the very early diagnosis of systemic sclerosis: results of a Delphi consensus study from EULAR scleroderma trials and research group. Ann Rheum Dis. 2011;70(3):476–81. 21081523 10.1136/ard.2010.136929

[9] Wollheim F. Classification of systemic sclerosis. Visions and reality. Rheumatology. 2005;44(10):1212–6. 15870151 10.1093/rheumatology/keh671

[10] LeRoy EC, Medsger TA Jr. Criteria for the classification of early systemic sclerosis. J Rheumatol. 2001;28(7):1573–6. 11469464

[11] Hudson M, et al. Quality of life in patients with systemic sclerosis compared to the general population and patients with other chronic conditions. J Rheumatol. 2009;36(4):768–72. 19228662 10.3899/jrheum.080281

[12] Almeida C, Almeida I, Vasconcelos C. Quality of life in systemic sclerosis. Autoimmun Rev. 2015;14(12):1087–96. 26212726 10.1016/j.autrev.2015.07.012

[13] Khanna D, et al. Abatacept in early diffuse cutaneous systemic sclerosis: results of a phase II investigator-initiated, multicenter, double-blind, randomized, placebo-controlled trial. Arthritis Rheumatol. 2020;72(1):125–36. 31342624 10.1002/art.41055 PMC6935399

[14] Khanna D, et al. Tocilizumab in systemic sclerosis: a randomised, double-blind, placebo-controlled, phase 3 trial. Lancet Respir Med. 2020;8(10):963–74. 32866440 10.1016/S2213-2600(20)30318-0

[15] Khanna D, et al. New composite endpoint in early diffuse cutaneous systemic sclerosis: revisiting the provisional American College of Rheumatology Composite Response Index in Systemic Sclerosis. Ann Rheum Dis. 2021;80(5):641–50. 33257497 10.1136/annrheumdis-2020-219100 PMC10750249

[16] Food and drug administration (2022) patient-focused drug development: selecting, developing, or modifying fit-for-purpose clinical outcome assessments. Guidance for industry, food and drug administration staff, and other stakeholders

[17] Volkmann ER, McMahan Z. Gastrointestinal involvement in systemic sclerosis: pathogenesis, assessment and treatment. Curr Opin Rheumatol. 2022;34(6):328–36. 35993874 10.1097/BOR.0000000000000899 PMC9547962

[18] Morrisroe KB, Nikpour M, Proudman SM. Musculoskeletal manifestations of systemic sclerosis. Rheum Dis Clin North Am. 2015;41(3):507–18. 26210132 10.1016/j.rdc.2015.04.011

[19] Kreps EO, et al. Ocular involvement in systemic sclerosis: a systematic literature review, it’s not all scleroderma that meets the eye. Semin Arthritis Rheum. 2019;49(1):119–25. 30660382 10.1016/j.semarthrit.2018.12.007

[20] Cheng KKF, Clark AM. Qualitative Methods and Patient-Reported Outcomes. Int J Qual Methods. 2017;16(1):1609406917702983.

[21] Johnson SR, O’Brien KK. Qualitative methods in systemic sclerosis research. J Rheumatol. 2016;43(7):1265–7. 27371646 10.3899/jrheum.160602

