# Supplementary material for: Development of a conceptual model of early systemic sclerosis (scleroderma)
Source: Orphanet J Rare Dis. 2025 Aug 19;20:446. doi: 10.1186/s13023-025-03917-8 (PMC12366407; doi:10.1186/s13023-025-03917-8)
Supplement: Supplementary file 1 — Additional file 1. [file 13023_2025_3917_MOESM1_ESM.docx]

# Supplementary Information

## Targeted literature review

Supplementary Table S1. United States and European clinical trials database search strategy

| Database search strategy | |
| --- | --- |
| Databases | clinicaltrials.gov, clinicaltrialsregister.eu |
| Trial dates | 01 January 2010–28 January 2020 |
| Phase | II, III and IV |
| Population | Diffuse cutaneous systemic sclerosis (dcSSc), Systemic sclerosis (SSc), and scleroderma |
| Other restrictions | English language |
| **Screening** |  |
| Inclusion criteria | Trials focused on patients with dcSSc and SSc and mentioning the use of a COA measure to assess symptoms and impacts of dcSSc and SSc |
| Exclusion criteria | Trials not including the name of the COA measure used |

Abbreviations: COA, clinical outcomes assessment; dcSSc, diffuse cutaneous systemic sclerosis; SSc, cutaneous systemic sclerosis.

Supplementary Table S2. Ovid Algorithm-Medline/Embase/Cochrane

| Search Number | Search Terms | Results |
| --- | --- | --- |
| **Disease State** | | |
| 1 | (diffuse cutaneous systemic sclerosis or dcSSc).ti. | 337 |
| 1 | *diffuse scleroderma/ or *Scleroderma, Diffuse/ or (systemic sclerosis or scleroderma or dcSSc).ti. | 41788 |
| **PRO/Questionnaires** | | |
| 2 | ((Patient or participant or subject or caregiver or family) adj3 (report$ or experience$ or assessment)).ti. | 52465 |
| 3 | *Patient Preference/ or (preference or preferences or acceptable risk or acceptable benefit or marginal rate of substitute or attribute importance or person trade off or PTO or willingness to pay or WTP or WTW).ti. | 67037 |
| 4 | *Choice Behavior/ or (discrete choice or discrete-choice or conjoint analysis or multi criteria decision analysis or multicriteria decision analysis or best worst scaling or best-worst scaling or benefit risk or thresholding or choice experiment or Swing weighting).ti. | 74825 |
| 5 | exp *Interviews as Topic/ or exp *interview/ or exp *questionnaire/ or exp *"Surveys and Questionnaires"/ or exp *rating scale/ or (interview$ or questionnaire$ or instrument$ or scale$ or inventor$ or score$ or survey or tool).ti. | 1113881 |
| 6 | (self-assess$ or self-complet$ or self-eval$ or reported-outcome or patient-rat$ or patient-reported or PROM or PREM or self-eval$ or patient-centered outcome$ or functional status or functional assessment or outcome measure$ or outcomes measure$ or outcome research or outcomes research or outcome assessment$ or outcomes assessment$ or self-report$ and patient-rat$ and patient-interview and patient assessment and patient-based-rating and patient-completed and evaluation stud$ and health status and health outcomes and outcome study or outcomes stud$ or disease burden or burden of illness).ti. or patient-based outcome$.ti,ab. | 7040 |
| 7 | exp *outcomes research/ or exp *"Outcome Assessment (Health Care)"/ or exp *outcome assessment/ or exp *Patient Reported Outcome Measures/ or exp *patient-reported outcome/ or exp *functional status/ or exp *functional assessment/ | 99839 |
| 8 | *Patient Satisfaction/ or (patient perception or treatment perception or patient reported outcome or patient-reported outcome or patient satisfaction or treatment satisfaction or treatment importance' or patient rated or patient preference$ or treatment priorit$ or impact of disease or disease impact or treatment theme or belief$).ti. | 10771 |
| 9 | exp *"Quality of Life"/ or exp *quality of life/ or (quality-of-life or QOL or HRQOL or hrql or hrqol or hr-qol or qol or mobility or Impact or function or functioning or burden or productivity or absenteeism or work or stigma).ti. | 1838116 |
| 10 | ((physical or psychologic$ or social or emotion$) adj3 (impact or outcome$ or status or assessment or impairment$ or deficit$)).ti. or ((Patient or participant or subject) adj3 (report$ or experience$)).ti. | 70708 |
| 11 | or/2-10 | 3203366 |
| 12 | 1 and 11 | 2643 |
| **Limits** | | |
| 13 | limit 12 to English language | 2489 |
| 14 | limit 13 to yr="2012 -09 March 2020" | 1543 |
| 15 | 14 not (animals/ not humans/) | 1540 |
| 16 | 14 not ((exp animal/ or nonhuman/) not exp human/) | 1525 |
| 17 | 15 or 16 | 1540 |
| 18 | (case report or case series or woman or man or child or adolescent or female or male or boy or girl or infant).ti. or exp case report/ or exp case study/ or case report$.jn. or case report$.jx. | 5513194 |
| 19 | (Congress or Ephemera or "Meeting Abstract" or "Introductory Journal Article" or News or "Newspaper Article" or Biography or Editorial or Comment or Letter or Overall).pt. or (commentary or editorial or comment or mouse or mice or rat or rats or animal).ti. | 7032110 |
| 20 | in vitro study/ or In Vitro Techniques/ | 1661238 |
| 21 | 17 not (10 or 19 or 20) | 1449 |
| 22 | Remove duplicates from 21 | 1003 |
| 23 | Limit 22 to (article or article in press) | **426** |

Abbreviations: dcSSc, diffuse cutaneous systemic sclerosis; HRQOL, health-related quality of life; PREM, patient-reported experience measure; PROM, patient-reported outcome measure; PTO, person trade-off; QOL, quality of life; SSc, cutaneous systemic sclerosis; WTP, willingness to pay.

Supplementary Table S3. Ovid Algorithm-PsychINFO

| Search Number | Search Terms | Results |
| --- | --- | --- |
| **Disease State** | | |
| 1 | (diffuse cutaneous systemic sclerosis or dcSSc).ti. | 0 |
| 2 | (systemic sclerosis or scleroderma or dcSSc) title  Limit to English and Journals  Limit to 2012–current | **46** |

Abbreviation: dcSSc, diffuse cutaneous systemic sclerosis.

Supplementary Table S4. Clinical sites

| Clinical Site | Location |
| --- | --- |
| Altoona Center for Clinical Research | Altoona, PA |
| Johns Hopkins University | Baltimore, MD |
| Georgetown University | Washington, DC |
| University of Michigan | Ann Arbor, MI |
| University of Pennsylvania | Philadelphia, PA |
| AZ Arthritis & Rheumatology Associates | Phoenix, AZ |
| Integrative Rheumatology of South Texas | Harlingen, TX |
| Space Coast Rheumatology & Arthritis | Merritt Island, FL |
| Family Arthritis Center | Palm Beach County, FL |
| Sun Valley Arthritis Center | Peoria, AZ |

Supplementary Table S5. Clinical outcomes assessment instruments identified via the clinical trials search and review of peer-reviewed literature

| COA Short Name |  | COA Type | Generic/Disease-specific | Number of Items | Concepts Measured |
| --- | --- | --- | --- | --- | --- |
| 6MWT | Six-minute Walk Test | ObsRO | Generic | NA | Mobility, exercise tolerance |
| AI VAS | Aesthetic Impairment VAS | PRO | Generic | 1 | View of severity of aesthetic severity |
| BDI | Beck Depression Inventory | PRO | Generic (Depression) | 21 | Sadness (1 item), discouraged about future (1 item), failure (1 item), satisfaction (1 item), guilt and punishment (3 items), self-worth (1 item), suicidality (1 item), crying (1 item), irritation (1 item), interest in others (1 item), decision-making (1 item), appearance (1 item), work (1 item), sleep and tiredness (2 items), weight and appetite (2 items), worry (1 item), sex drive/libido (1 item) |
| BPI | Brief Pain Inventory | PRO | Generic (Pain) |  | Worst pain in last 24 hours, general activity, least pain in last 24 hours, mood, pain on average, walking ability, pain right now, normal work (including housework), relations with other people, sleep, enjoyment of life |
| Cattell Anxiety Self-assessment Scale | Cattell Anxiety Self-assessment Scale | PRO | Generic (Anxiety) | 40 | Anxiety |
| CES-D | Center for Epidemiologic Studies Depression Scale | PRO | Generic (Depression) | 20 | Depression |
| *CHFDS* | *Cochin Hand Function Scale* | *PRO* | *Osteoarthritis* | *18* | *Functional ability in the hand. Five subscales: kitchen, dressing, hygiene, office, and other* |
| COPE | Coping Orientation to Problems Experienced | PRO | Generic | 60 | Positive reinterpretation and growth (4 items), mental disengagement (4 items), focus on and venting of emotions (4 items), use of instrumental social support (4 items), active coping (4 items), denial (4 items), religious coping (4 items), humor (4 items), behavioral disengagement (4 items), restraint (4 items), use of emotional social support (4 items), substance use (4 items), acceptance (4 items), suppression of competing activities (4 items), planning (4 items) |
| *CRISS* | *Combined Response Index for Systemic Sclerosis* | *ClinRO* | *Specific to SSc* | *Composite score* | *mRSS, forced vital capacity predicted, HAQ-DI, Patient Global Assessment, Physician Global Assessment* |
| *DAS28* | *Disease Activity Score* | *ClinRO* | *Specific to rheumatoid arthritis/musculoskeletal indications* | *Composite score* | *Quantifies abnormalities in patients with rheumatoid arthritis. The swollen joint count reflects the amount of inflamed synovial tissue and the tender joint count is associated with the level of pain.* |
| DEQ | Dry Eye Questionnaire | PRO | Generic (dry eye) | 4 | Degree of irritation, frequency, intensity in the morning, and intensity late in the day |
| EQ-5D | EQ-5D | PRO | Generic (QoL) | 5 | Mobility, looking after myself, doing usual activities, having pain or discomfort, and feeling worried, sad, or unhappy |
| FACIT-Fatigue | Functional Assessment of Chronic Illness Therapy Fatigue Scale | PRO | Generic (QoL) | 40 | Physical well-being (7 items), social/family well-being (7 items), emotional well-being (6 items), functional well-being (7 items) and additional concerns (13 items) |
| FSFI | Female Sexual Function Index | PRO | Generic (sexual functioning) | 19 | Desire (2 items), arousal (4 items), lubrication (4 items), orgasm (3 items), satisfaction (3 items), pain (3 items) |
| FSS | Fatigue Severity Scale | PRO | Generic (fatigue) | 9 | Motivation (1 item), triggers (2 items), physical functioning (5 items), family/social life (1 item) |
| HADS | Hospital Anxiety and Depression Scale | PRO | Generic (anxiety and depression) | 14 | Anxiety (7 items) and depression (7 items) |
| HAMIS | Hand Mobility in Scleroderma test | PRO | Specific to SSc | 5 | One item for each: finger flexion and extension, abduction of the thumb, pincer grip, finger abduction/swelling, dorsal extension and volar flexion of the wrist; pronation (4 items) and supination (4 items) |
| *HAQ-DI* | *Health Assessment Questionnaire Disability Index* | *PRO* | *Generic* | *21 + 2 on aids/devices* | *Dressing/grooming, arising, eating, walking, hygiene, reach, grip, activities, 1 VAS pain item 0–100* |
| *HAQ* | *Health Assessment Questionnaire* | *PRO* | *Generic and musculoskeletal diseases* | *21 + 2 on aids/devices* | *Dressing/grooming, arising, eating, walking, hygiene, reach, grip, activities, 1 VAS pain item 0–100* |
| HDISS* | Hand Disability in Systemic Sclerosis Digital Ulcers | PRO | Specific to SSc | 24 | Hand mobility: hold a plate, pour liquid, unscrew lid, cut food, use fork, prepare food, wash dishes, button clothing, open/close zipper, pull on socks, tie shoelaces, hold toothbrush, wash hands, shower or bath, brush hair, put cream on face, write short sentence, type on keyboard, press buttons, press cell phone, turn a key, use scissors, pick up coins, sweep floor |
| IIEF-5 | International Index for Erectile Function-5 | PRO | Generic (sexual functioning) | 15 | Sexual activity (6 items), sexual intercourse satisfaction (3 items), sexual desire (2 items), overall satisfaction (2 items), orgasmic function (2 items) |
| IPQ-R | Illness Perception Questionnaire Revised | PRO | Generic | 70 | Symptoms experienced (14 items), views about illness (38 items), possible causes (18 items) |
| Itch NRS | Itch Numeric Rating Scale | PRO | Generic (skin itching) | 1 | Itch severity over past 24 hours |
| LANSS | Leeds Assessment of Neuropathic Symptoms and Signs | ClinRO and PRO | Generic (pain) | 7 | Pain sensation (1 item), appearance of painful skin (1 item), skin sensitivity to touch (1 item), pain occurrence (1 item), skin temperature (1 item), pain experience (2 items) |
| MACTAR | McMaster Toronto Arthritis Patient Preference Disability Questionnaire | PRO | Arthritis | NR | General health, physical function, social function, and emotional function |
| McMonnies Questionnaire | McMonnies Questionnaire | PRO | Generic (dry eye) | 14 | One item for each, unless noted otherwise: Previous treatment of dry eye, experience of symptoms (2 items), frequency of symptoms, unusual sensitivity of eyes, swimming irritation of eyes, alcohol use, medication side effects, arthritis, mucous membrane dryness, thyroid abnormality, nocturnal lagophthalmos, waking irritation |
| MHISS | Mouth Handicap in Systemic Sclerosis Scale | PRO | Specific to SSc | 12 | Reduced mouth opening (5 items), sicca syndrome (5 items), aesthetic concerns (2 items) |
| MMSE | Mini-Mental State Examination | ClinRO | Cognitive function | 30 | Orientation (10 items), registration (3 items), attention/calculation (5 items), recall (3 items), language (8 items), copying (1 item) |
| *MMT* | *Manual Muscle Testing* | *ClinRO* | *Generic (multiple indications)* | *Clinician composite score* | *Function of the muscle (Score 1), movement in horizontal plane (Scores 1–2); antigravity position (Scores 3–10)* |
| MPQ | McGill Pain Questionnaire | PRO | Generic (pain) | 15 (short form) 72 (full) | Pain sensation, triggers, and severity |
| *mRSS* | *Modified Rodnan Skin Score* | *ClinRO* | *Specific to SSc* | *5* | *Skin thickening by clinician palpation* |
| *MSSª* | *Medsger Severity Scale* | *ClinRO* | *Specific to SSc* | *9* | *General health, peripheral vascular, skin, joint/tendon, muscle, GI, lungs, heart, kidney* |
| NYHA | New York Heart Association Scale | ClinRO | Generic | NA | Physical activity limitations, breathing, angina pain |
| OSDI | Ocular Surface Disease Index | PRO | Generic (eye diseases) | 12 | Light sensitivity (1 item), sore/painful eyes (2 items), blurred or poor vision (2 items), reading (1 item), nighttime driving (1 item), working at a computer (1 item), watching TV (1 item), environmental triggers (3 items) |
| PDSBE | Disability Sexual and Body Esteem Scale | PRO | Generic | 3 | View of self and body |
| *PGA (Patient)* | *Patient Global Assessment* | *PRO* | *Generic* | *1* | *Global functioning* |
| *PGA (Physician)* | *Physician Global Assessment* | *ClinRO* | *Generic* | *1* | *Global functioning* |
| POMS | Profile of Mood States | PRO | Generic | 65- and 35-item versions | Mood (negative subscales: tension, depression, fatigue, confusion, anger; positive subscale: vigor and esteem-related affect). |
| *PROMIS-29* | *Patient-Reported Outcomes Measurement Information System - 29 Profile V2.1* | *PRO* | *Generic* | *29* | *Seven domains: physical function, fatigue, pain interference, depressive symptoms, anxiety, ability to participate in social roles and activities, and sleep disturbance* |
| PROMIS-Sleep | Patient-Reported Outcomes Measurement Information System - Sleep | PRO | Generic (sleep) | 27 | Qualitative aspects of sleeping (sleep initiation and maintenance) and wakeful functioning |
| *PROMIS-Fatigue* | *Patient-Reported Outcomes Measurement Information System - Fatigue* | *PRO* | *Generic (fatigue)* | *4* | *Fatigue-only domain (4 items)* |
| PSQI | Pittsburgh Sleep Quality Index | PRO | Generic (sleep) | 10 | Time of sleep, time taken to fall asleep, time of waking up, hours of actual sleep per night, trouble sleeping (10 items), over the counter sleep medications, difficulty staying awake during daytime, enthusiasm, sleep quality, sleep arrangements (6 items) |
| Rosenberg Self-esteem Scale | Rosenberg Self-esteem Scale | PRO | Generic | 10 | Positive (5 items) and negative (5 items) opinions of self |
| *SF-36ª* | *36-item Short Form Health Survey* | *PRO* | *Generic (QoL)* | *36* | *Eight domains: Physical functioning (10 items), bodily pain (2 items), role limitations due to physical health perceptions (4 items), general health perceptions (5 items), mental health (5 items), role limitations due to emotional problems (3 items), vitality (4 items), social functioning (2 items), and health transition (1 item)* |
| *SGRQ* | *St George’s Respiratory Questionnaire* | *PRO* | *Respiratory tract and immune system diseases* | *50* | *Airflow limitation, symptoms component (frequency and severity), activities that cause or are limited by breathlessness, impact components (social functioning, psychological disturbances resulting from airways disease)* |
| *SHAQ-DI* | *Scleroderma Health Assessment Questionnaire Disability Index* | *PRO* | *Specific to SSc* | *19 + 2 about aids and devices* | *Composed of the HAQ and VAS severity: Dressing/grooming, arising, eating, walking, hygiene, reach, grip, activities, six VAS items 0–100* |
| SScQoL | SSc Quality of Life Questionnaire | PRO | Specific to SSc | 29 | Emotion, physical adaptation, impact on/with others, and impact on self |
| *SSPRO* | *Scleroderma Skin Patient-reported Outcome* | *PRO* | *Specific to SSc* | *18* | *Physical symptoms (5 items), emotional effects (6 items), physical function (4 items), social effects (3 items)* |
| *SSQ* | *Short form of social support* | *PRO* | *Generic* | *6* | *Social support (people supporting them, satisfaction with support)* |
| *TDI (BDI-TDI)* | *Baseline and Transition Dyspnea Indexes* | *ClinRO* | *Respiratory: Asthma, cystic fibrosis, Chronic obstructive pulmonary disease* | *1* | *Shortness of breath* |
| *UCLA SCTC-GIT Composite* | *UCLA Scleroderma Clinical Trial Consortium Gastrointestinal Tract Instrument* | *PRO* | *Gastrointestinal Symptoms* | *52  Revised: 34* | *Reflux/indigestion, diarrhea, constipation, pain, emotional well‐being, and social functioning* |
| *VAS* | *Visual Analog Scale* | *PRO* | *Generic* | *1* | *Overall health* |
| WALS | Workplace Activity Limitation Scale | PRO | Generic | 11- and 12-item versions | Mobility, prolonged sitting and standing, lifting, working with hands, crouching, bending or kneeling, reaching, scheduling, work hours, pace of work, concentration, and meeting current job demands. An item asking about difficulties concentrating on work is not included in the 11‐item version |
| WHOQoL | World Health Quality of Life-Brief Version | PRO | Generic | 26 | Physical health (7 items), psychological (6 items), social relationships (3 items), environment (8 items) |
| WoC | Ways of coping | PRO | Generic | 68 | Coping with life stressors |
| WPAI | Work Productivity and Activity Impairment Questionnaire | PRO | Generic | 9 | Presenteeism, absenteeism, productivity loss, activity impairment |

Italics indicate the 19 COAs identified via the clinical trials search. Abbreviations: ClinRO, clinician-reported outcome; COA, clinical outcomes assessment; GI, gastrointestinal; HAQ-DI, Health Assessment Questionnaire Disability Index; NA, not applicable; NR, not reported; ObsRO, observer-reported outcome; PRO, patient-reported outcome; QoL, quality of life; SSc, cutaneous systemic sclerosis; VAS, visual analog scale.

* An article not captured by the search due to its EMBASE indexing status was suggested by the study sponsor (CSL Behring) and included after screening.

ª Materials identified via both clinical trials search and peer-reviewed literature review.

Supplementary Table S6. Participant-reported systemic sclerosis symptoms

| Symptoms, n (%) | Overall (n=44) | dcSSc (n=32) | lcSSc (n=12) |
| --- | --- | --- | --- |
| **Hand symptoms** | **44 (100.0)** | **32 (100)** | **12 (100)** |
| Raynaud's phenomenon/cold intolerance | 42 (95.5) | 30 (93.8) | 12 (100.0) |
| *Loss of grip strength* | 31 (70.5) | 25 (78.1) | 6 (50.0) |
| *Loss of hand function* | 27 (61.4) | 23 (78.1) | 4 (33.3) |
| Puffy fingers | 22 (50.0) | 15 (46.9) | 7 (58.3) |
| Stiffness of hands | 20 (45.5) | 16 (50.0) | 4 (33.3) |
| Nailfold capillary changes | 17 (38.6) | 15 (46.9) | 2 (16.7) |
| Swollen hands | 14 (31.8) | 12 (37.5) | 2 (16.7) |
| Digital ulcers | 10 (22.7) | 8 (25.0) | 2 (16.7) |
| *Amputation* | 2 (4.5) | 1 (3.1) | 1 (8.3) |
| **Skin symptoms** | **44 (100.0)** | **32 (100)** | **12 (100)** |
| Skin fibrosis | 36 (81.8) | 30 (93.8) | 6 (50.0) |
| Skin color change | 32 (72.7) | 24 (75.0) | 8 (66.7) |
| Skin itchiness | 31 (70.5) | 25 (78.1) | 6 (50.0) |
| Skin swelling | 24 (54.5) | 19 (59.4) | 5 (41.7) |
| *Dry skin* | 10 (22.7) | 4 (12.5) | 6 (50.0) |
| *Red skin* | 9 (20.5) | 5 (15.6) | 4 (33.3) |
| Calcium nodules | 8 (18.2) | 6 (18.8) | 2 (16.7) |
| Dilated blood vessels | 8 (18.2) | 5 (15.6) | 3 (25.0) |
| Open sores | 5 (11.4) | 5 (15.6) | 0 |
| Ulcers | 3 (6.8) | 3 (9.4) | 0 |
| Vascular lesions | 2 (4.5) | 1 (3.1) | 1 (8.3) |
| *Easy bruising* | 1 (2.3) | 1 (3.1) | 0 |
| **Gastrointestinal symptoms** | **42 (95.5)** | **30 (93.8)** | **12 (100)** |
| Heartburn/Reflux | 37 (84.1) | 27 (84.4) | 10 (83.3) |
| Difficulty swallowing | 22 (50.0) | 14 (43.8) | 8 (66.7) |
| Diarrhea | 20 (45.5) | 16 (50.0) | 4 (33.3) |
| Distention/bloating | 18 (41.0) | 13 (40.6) | 5 (41.7) |
| Constipation | 14 (31.8) | 9 (28.1) | 5 (41.7) |
| Nausea | 12 (27.3) | 11 (34.4) | 1 (8.3) |
| Vomiting | 10 (22.7) | 6 (18.8) | 4 (33.3) |
| *Gas* | 3 (6.8) | 2 (6.3) | 1 (8.3) |
| *Gastrointestinal bleeding* | 2 (4.5) | 2 (6.3) | 0 |
| *Early satiety* | 2 (4.5) | 2 (6.3) | 0 |
| **Pain symptoms** | **42 (95.5)** | **31 (96.9)** | **11 (91.7)** |
| Joint pain | 32 (72.7) | 25 (78.1) | 7 (58.3) |
| Hand pain | 25 (56.8) | 18 (56.3) | 7 (58.3) |
| Muscle pain | 21 (47.7) | 19 (59.4) | 2 (16.7) |
| Neuropathic pain | 12 (27.3) | 7 (21.9) | 5 (41.7) |
| Abdominal pain | 11 (25.0) | 9 (28.1) | 2 (16.7) |
| Skin pain | 6 (13.6) | 5 (15.6) | 1 (8.3) |
| *Foot pain* | 6 (13.6) | 4 (12.5) | 2 (16.7) |
| Pelvic pain | 3 (6.8) | 2 (6.3) | 1 (8.3) |
| Discomfort | 2 (4.5) | 2 (6.3) | 0 |
| *Headaches* | 2 (4.5) | 2 (6.3) | 0 |
| **Mouth symptoms** | **36 (81.8)** | **30 (93.8)** | **6 (50.0)** |
| Dry mouth | 23 (52.3) | 17 (53.1) | 6 (50.0) |
| Difficulty opening mouth | 19 (43.2) | 18 (56.3) | 1 (8.3) |
| Reduction in size of mouth | 12 (27.3) | 11 (34.4) | 1 (8.3) |
| *Numbness in mouth* | 2 (4.5) | 1 (3.1) | 1 (8.3) |
| *Mouth sores* | 2 (4.5) | 2 (6.3) | 0 |
| *Tongue tightness* | 2 (4.5) | 2 (6.3) | 0 |
| **Joint symptoms** | **35 (79.5)** | **28 (87.5)** | **7 (58.3)** |
| Stiffness of joints | 20 (45.5) | 16 (50.0) | 4 (33.3) |
| Arthritis | 18 (40.9) | 14 (43.8) | 4 (33.3) |
| Joint contracture | 14 (31.8) | 11 (34.4) | 3 (25.0) |
| Tender joints | 7 (15.9) | 6 (18.8) | 1 (8.3) |
| Swollen joints | 11 (25.0) | 9 (28.1) | 2 (16.7) |
| **Muscle symptoms** | **31 (70.5)** | **24 (75.0)** | **7 (58.3)** |
| Muscle weakness | 25 (56.8) | 20 (62.5) | 5 (41.7) |
| Muscle tenderness | 6 (13.6) | 4 (12.5) | 2 (16.7) |
| *Muscle tightness* | 3 (6.8) | 3 (9.4) | 0 |
| *Tendon tightness* | 2 (4.5) | 2 (6.3) | 0 |
| *Muscle cramps* | 1 (2.3) | 0 | 1 (8.3) |
| *Tremors* | 1 (2.3) | 0 | 1 (8.3) |
| *Muscle atrophy* | 1 (2.3) | 1 (3.1) | 0 |
| **Ocular symptoms** | **22 (50.0)** | **17 (53.1)** | **5 (41.7)** |
| *Dry eye* | 8 (19.2) | 8 (25.0) | 0 |
| Eye discomfort | 7 (15.9) | 5 (15.6) | 2 (16.7) |
| *Sensitivity to light* | 6 (13.6) | 6 (18.8) | 0 |
| *Vision impairment* | 6 (13.6) | 5 (15.6) | 1 (8.3) |
| Eyelid inflammation | 3 (6.8) | 0 | 3 (25.0) |
| **Cognitive symptoms** | **21 (47.7)** | **17 (53.1)** | **4 (33.3)** |
| Difficulty remembering | 13 (29.5) | 10 (31.3) | 3 (25.0) |
| Difficulty concentrating | 10 (22.7) | 10 (31.3) | 0 |
| *Brain fog* | 5 (11.4) | 4 (12.5) | 1 (8.3) |
| Attention | 4 (9.1) | 4 (12.5) | 0 |
| Recall | 4 (9.1) | 2 (6.3) | 2 (16.7) |
| Language | 3 (6.8) | 2 (6.3) | 1 (8.3) |
| Orientation | 1 (2.3) | 1 (3.1) | 0 |
| **Lung symptoms** | **20 (45.5)** | **15 (46.9)** | **5 (41.7)** |
| Dyspnea | 16 (36.4) | 13 (40.6) | 3 (25.0) |
| Persistent cough | 8 (18.2) | 5 (15.6) | 3 (25.0) |
| Lung involvement | 6 (13.6) | 6 (18.8) | 0 |
| Pulmonary hypertension | 4 (9.1) | 2 (6.3) | 2 (16.7) |
| Interstitial lung disease | 6 (13.6) | 5 (15.6) | 1 (8.3) |
| **Sexual symptoms** | **20 (45.5)** | **18 (56.3)** | **2 (16.7)** |
| Lack of desire | 11 (25.0) | 9 (28.1) | 2 (16.7) |
| Vaginal dryness | 10 (22.7) | 8 (25.0) | 2 (16.7) |
| Sexual dysfunction | 4 (9.1) | 3 (9.4) | 1 (8.3) |
| Impaired arousal | 3 (6.8) | 2 (6.3) | 1 (8.3) |
| Impaired orgasm | 3 (6.8) | 2 (6.3) | 1 (8.3) |
| Erectile dysfunction | 3 (6.8) | 3 (9.4) | 0 |
| Dyspareunia | 1 (2.3) | 0 | 1 (8.3) |
| **Cardiac symptoms** | **13 (29.5)** | **11 (34.4)** | **2 (16.7)** |
| Edema | 9 (20.5) | 8 (25.0) | 1 (8.3) |
| Irregular heartbeat | 4 (9.1) | 3 (9.4) | 1 (8.3) |
| Cardiac complications | 3 (6.8) | 3 (9.4) | 0 |
| Swelling around heart | 2 (4.5) | 2 (6.3) | 0 |
| **Miscellaneous symptoms** |  |  |  |
| Fatigue | 35 (79.5) | 28 (87.5) | 7 (58.3) |
| Weight loss | 22 (50.0) | 20 (62.5) | 2 (16.7) |
| Changes in appearance | 20 (45.5) | 18 (56.3) | 2 (16.7) |
| Debilitating physical changes | 9 (20.5) | 8 (25.0) | 1 (8.3) |
| *Poor circulation in arms* | 2 (4.5) | 1 (3.1) | 1 (8.3) |
| *Numbness in extremities* | 1 (2.3) | 0 | 1 (8.3) |
| Renal crisis | 1 (2.3) | 1 (3.1) | 0 |

Italics indicate symptoms reported during the concept elicitation interviews that had not previously been identified in pertinent literature. Abbreviations: dcSSc, diffuse cutaneous systemic sclerosis; lcSSc, limited cutaneous systemic sclerosis.

Supplementary Table S7. Participant quotes for the most frequently reported SSc symptoms across subtypes

| Symptom | Illustrative Patient Quotes |
| --- | --- |
| **Hand symptoms** |  |
| Raynaud's phenomenon/cold intolerance | “One of the first symptoms I noticed was my Raynaud's, when my fingertips would turn purple.”  *014-001 (dcSSc)*  “My tongue, if I talk too much, I try to avoid talking when it’s cold because my tongue, like half of it goes white and it loses – you won’t understand what I’m telling you.”  *014-010 (lcSSc)* |
| Loss of grip strength | “Well, I can still grip it, I just don't have the strength to grip it. (…) I cut wire with pliers (…) the larger size wire is just harder to cut. I used to be able to cut it a few years ago and now I don't have the strength to cut it.”  *013-008 (lcSSc)* |
| Loss of hand function | “ (…) there’s some things that I just can’t do anymore. Like we go fishing, we have boat, we put it out on a lake, I catch a fish, I’d start reeling it in. If it’s way out there fighting me, I can’t get it in.”  *001-004 (dcSSc)* |
| Puffy fingers | “They look sort of puffy compared to what they used to be. (…) They just look sort of bent all the time. Like, I can't extend them all the way flat. So, they look kind of curled, you know, but they're not. They're thicker, but I don't think it's from swelling at the joints. I think it's from the skin.”  *003-005 (dcSSc)* |
| **Skin symptoms** |  |
| Skin fibrosis | “My forearms, well, my arms, like from the elbow down is when I first started noticing it because it was so odd, I mean to touch my skin was like my skin was hard, tight. It felt like it was going to split open (…)”  *004-006 (dcSSc)* |
| Increase/decrease in pigmentation | “ (…) the top and bottom of my arm below my elbow, it’s just kind of red and mottled, and down by my wrist, there’s like a band of what I would call darker-looking skin (…)”  *004-006 (dcSSc)* |
| Skin itchiness | “As the skin continued to get thicker, that continued to happen more often with the itching.”  *002-002 (dcSSc)* |
| Skin swelling | “I have swelling in the morning when I get up, my hands are pretty severely swollen every day when I wake up, and as I walk or use any muscle at all, I get swelling. Like if was to repeatedly twist something, my arm would start bothering me.”  *001-006 (dcSSc)* |
| **Gastrointestinal symptoms** |  |
| Reflux | “My acid reflux has been a lot worse lately. I mean I used to only have to deal with it when I was laying down at night. Now it seems to be apparent even when I’m sitting down and straight up.”  *005-003 (dcSSc)* |
| Heartburn | “I would—I think I tend to use the terms [reflux and heartburn] interchangeably. The—are a bit different. Sometimes I have more of a traditional heartburn like a burning feeling.”  *002-003 (dcSSc)* |
| Difficulty swallowing | “I mean I can tolerate it, but I have a hard time sometimes when I’m eating and my throat will get bound up with food.”  *001-006 (dcSSc)* |
| **Pain symptoms** |  |
| Joint pain | “Yeah, knee joints, ankles, elbows, shoulders, anywhere there’s a joint, there was inflammation, stiffness, pain.”  *005-003 (dcSSc)* |
| Hand pain | “The pain in my hands I would say is some of the most difficult pain that I deal with. It had ever since the beginning been worse on my right hand, on my dominant hand. (…) It’s not only my fingers. It’ll kind of go down into the larger part of my hand, and within the last couple months has moved to my wrists.”  *002-003 (dcSSc)* |
| **Mouth symptoms** |  |
| Dry mouth | “(…) it happens frequently at night, and I’ll wake up and my mouth feels like the Sahara Desert, and even if I go to get a drink and rinse my mouth with a glass of water, it still just doesn't feel like it wants to come back to normal, and then there’s other times during the day when my mouth feels normal, and I’ll start going on my walk, the next thing I know, man, I’m just dry (…).”  *004-014 (dcSSc)* |
| **Joint symptoms** |  |
| Stiffness of joints | “My knees, ankles, I mean, pretty much every joint in my body, (…) at different points, is very stiff.”  *004-001 (dcSSc)* |
| **Muscle symptoms** |  |
| Muscle weakness | “My muscles are very weak. Certain things that I try to do, like say if I have to fold clothes and everything, picking them up and trying to fold them, they seem very heavy to me.”  *013-005 (dcSSc)* |
| **Ocular symptoms** |  |
| Dry eye | “I have a lot of dryness that has gotten worse ever since, and I feel at times that I have kind of like trouble focusing (…)”  *007-002 (dcSSc)* |
| **Cognitive symptoms** |  |
| Difficulty remembering | “Like I have left I don't know how many times stuff on the stove, and it’s on fire.”  *014-010 (lcSSc)* |
| **Lung symptoms** |  |
| Dyspnea | “I had to quit work because of it… I got too short of breath (…)”  *002-001 (lcSSc)* |
| **Sexual symptoms** |  |
| Lack of desire | “In terms of like, sex drive, I would say, yeah, sort of, but mostly because I'm like exhausted all the time, but not because of a lack of interest. It's just sort of like, well, I'm so tired.”  *003-005 (dcSSc)* |
| **Cardiac symptoms** |  |
| Irregular heartbeat | “(…) I told my cardiologist that at times I can be sitting, and I can feel my heart. I say, ‘I can feel it jumping off rhythm, and I feel it’s not normal.’”  *007-002 (dcSSc)* |
| **Miscellaneous symptoms** |  |
| Fatigue | “Whereas fatigue is more like I can't get out of bed. I just want to sleep. I mean, there's times when I'm in my office, and I am like staring at my computer, and I can just fall back and sleep in my chair, and that's what I want to do.”  *003-003 (dcSSc)* |
| Weight loss | “ Before I got diagnosed, I was 136 pounds. Now after X amount of months, I’m down to 100 pounds. And that includes weight and muscle, losing muscle mass.”  *002-006 (dcSSc)* |
| Changes in appearance | “(…) I started having bald spots, like real big bald spots.”  *004-012 (lcSSc)*  “No, I mean the tightness of the skin, shininess of the skin, my hands look a little weird because of the swelling of the fingers.”  *007-003 (dcSSc)* |

All symptoms affecting ≥50% of participants are illustrated with one quote each. Where a group of symptoms contains no single symptom affecting ≥50% of patients, the single most common symptom is illustrated with a quote. Patient identifiers are in italics. Abbreviations: dcSSc, diffuse cutaneous systemic sclerosis; lcSSc, limited cutaneous systemic sclerosis.


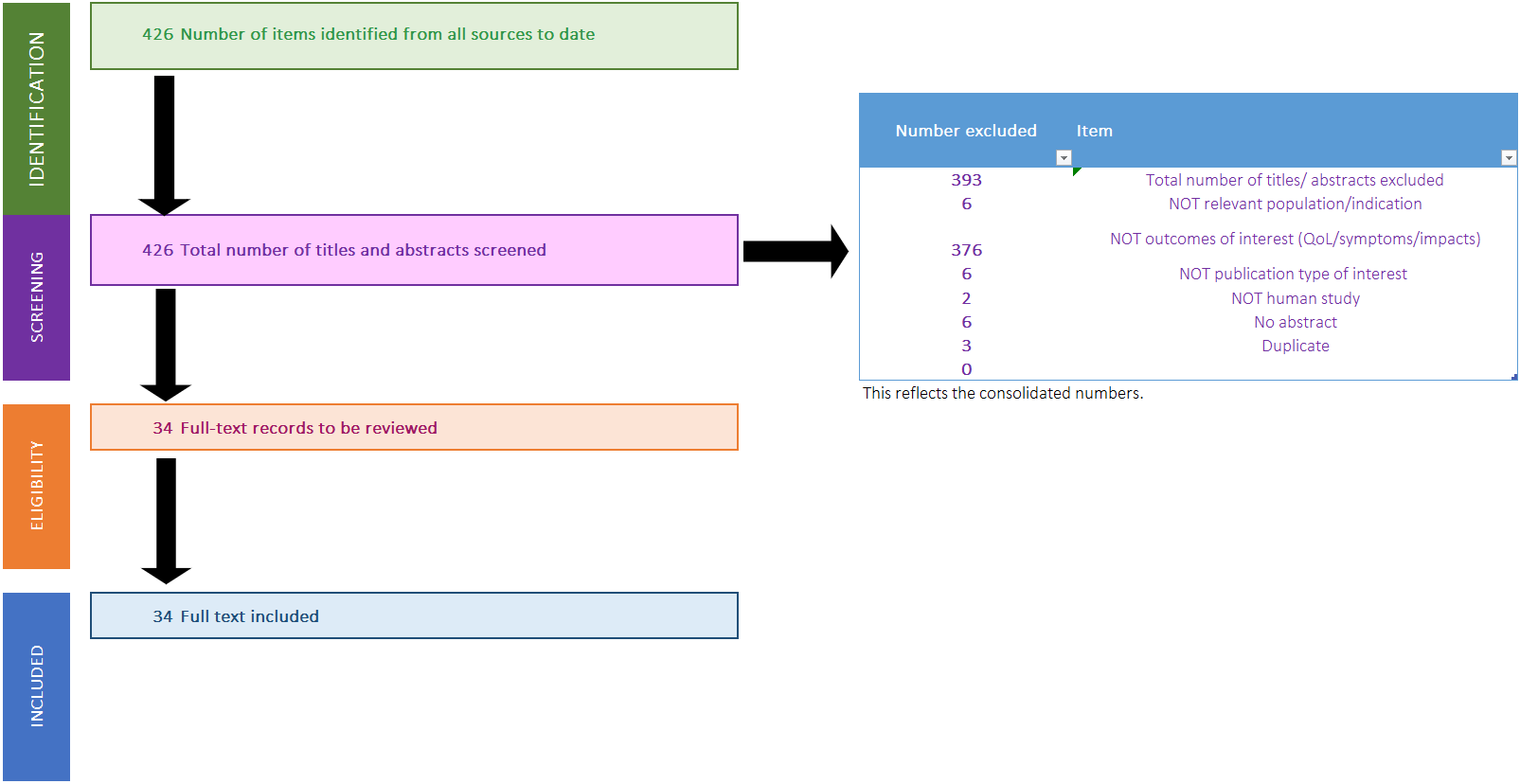


Supplementary Figure S1. Preferred Reporting Items for Systematic Reviews and Meta-Analyses (PRISMA) diagram. N=1 additional article was identified and included in the extraction. In total, 35 articles were included in the full-text extraction. Abbreviation: QoL, quality of life.


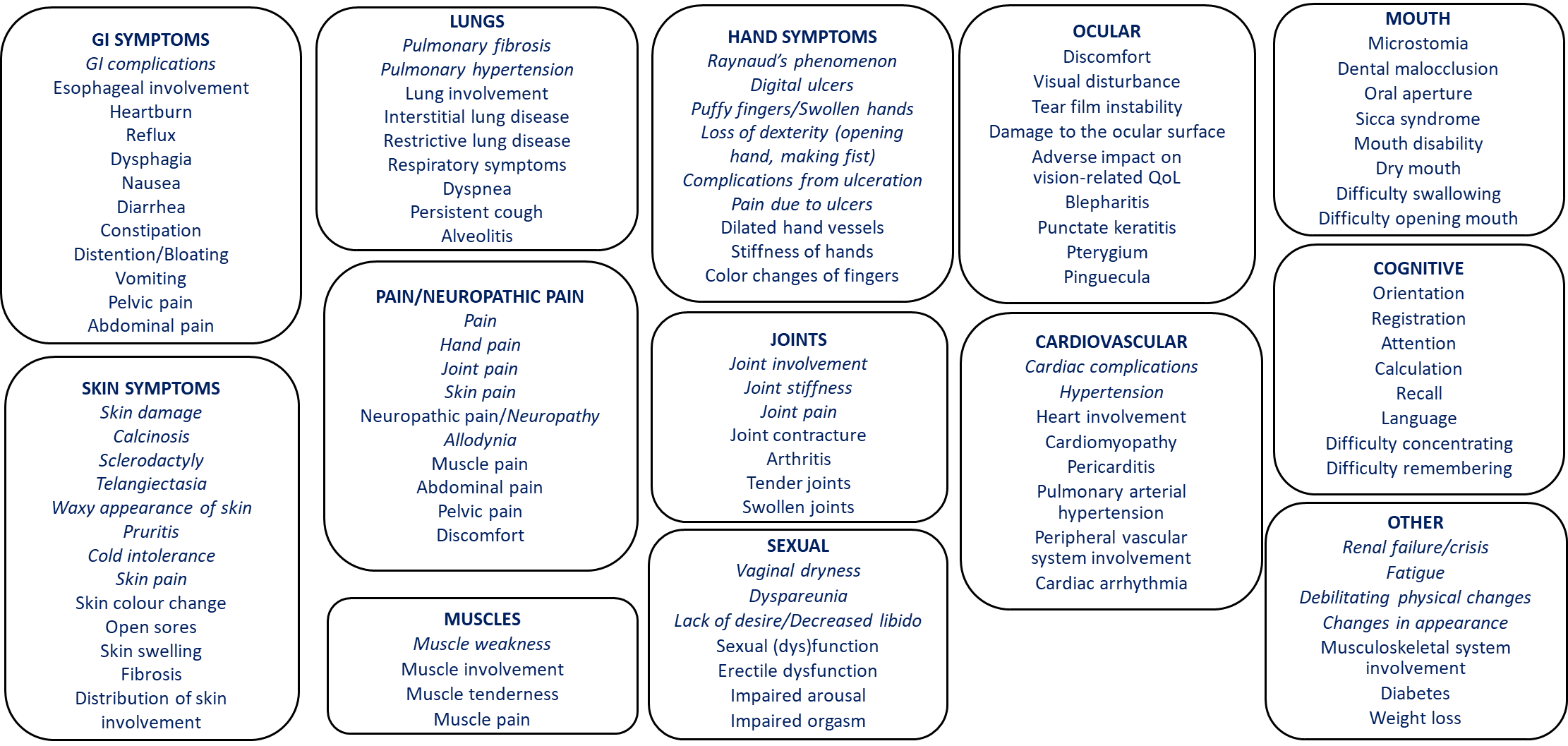


Supplementary Figure S2. Preliminary global conceptual model of physical symptoms in diffuse cutaneous systemic sclerosis. Text in italics identifies concepts extracted from publications (included in the targeted literature review) describing qualitative research. Abbreviations: GI, gastrointestinal; QoL, quality of life.
